# Intelligent smart sensing with ResNet-PCA and hybrid ML–DNN for sustainable and accurate plant disease detection

**DOI:** 10.3389/fpls.2025.1691415

**Published:** 2025-11-06

**Authors:** Shtwai Alsubai, Ahmad Almadhor, Abdullah Al Hejaili, Thippa Reddy Gadekallu

**Affiliations:** 1 College of Computer Engineering and Sciences, Prince Sattam bin Abdulaziz University, Al Kharj, Saudi Arabia; 2 Department of Computer Engineering and Networks, College of Computer and Information Sciences, Jouf University, Sakaka, Saudi Arabia; 3 Computer Science Department, Faculty of Computers & Information Technology, University of Tabuk, Tabuk, Saudi Arabia; 4 College of Mathematics and Computer Science, Zhejiang A&F University, Hangzhou, China; 5 College of Mechanical Engineering, Jiaxing University, Jiaxing, China; 6 Division of Research and Development, Lovely Professional University, Phagwara, India

**Keywords:** hybrid machine learning–deep learning, intelligent smart sensing, sustainable disease detection, ResNet feature extraction, Principal Component Analysis (PCA)

## Abstract

**Introduction:**

Diseases of plants remain one of the greatest threats to sustainable agriculture, with a direct adverse effect on crop productivity and threatening food security worldwide. Conventional detection methods rely heavily on manual detection and laboratory analysis, which are time-consuming, subjective, and unsuitable for large-scale monitoring. The use of the most recent progress in computer vision and artificial intelligence has opened up a prospect of automated, scalable, and precise disease diagnosis.

**Methods:**

This paper introduces a feature-efficient hybrid model that trains classical Machie Learning (ML) classifiers with Deep Neural Network (DNN) using ResNet-based feature extraction and Principal Component Analysis (PCA). The PlantVillage dataset with mixed crop-disease pairs is used to implement and thoroughly test five hybrid models.

**Results:**

Wide-ranging experiments proved that the Logistic Regression (LR)+DNN hybrid resulted in the best classification accuracy of 96.22% as compared to other models and available benchmarks. Besides being able to outperform other techniques in terms of predictive power, the framework displayed good training stability and robustness to class imbalance as well as a higher degree of interpretability based on LIME-based analysis.

**Discussion:**

The obtained results confirm the hybrid ML+DNN paradigm as a safe, transparent, scalable disease recognition framework when applied to plant diseases. Providing opportunities for timely and accurate disease detection, the proposed framework can help with precision agriculture, where pesticide use can be reduced, consequently, and a significant contribution to sustainable farming can be achieved.

## 1 Introduction

Plant diseases pose a serious threat to global agriculture, directly affecting crop productivity, food security, and the livelihoods of farmers (Gai and Wang, 2024). According to the Food and Agriculture Organization (FAO), crop losses due to plant pathogens and pests can reach as high as 40 percent worldwide, resulting in significant economic losses and threatening the sustainability of agricultural systems (Junaid and Gokce, 2024). The current approach to diagnosing these diseases relies heavily on manual examinations by trained professionals, which can be time-consuming, labor-intensive, and subjective, leading to inaccuracies, particularly in regions with a shortage of agronomists (Elangovan et al., 2024). This makes early and accurate diagnosis crucial for sustainable crop management and minimizing avoidable losses (Rajendiran and Rethnaraj, 2024). Inadequate diagnosis often leads to other issues, such as the overuse of pesticides, which can have detrimental effects on both environmental balance and soil quality (Shahbazi et al., 2025).

However, advancements in digital imaging technology and artificial intelligence (AI) have paved the way for automated plant disease detection (Negi and Anand, 2024). Traditional plant pathology methods, including physical symptom observation and lab-based diagnostics, while reliable, fall short for large-scale monitoring (Khakimov et al., 2022). In contrast, computer vision techniques leveraging ML and (DL) can rapidly and effectively identify disease patterns directly from leaf images (Upadhyay et al., 2025). Despite these advancements, research has predominantly focused on singular ML or DL models, which can be limited in their feature extraction abilities and may lack robustness when analyzing disease trends across different crop species. Although deep learning models such as ResNet, VGG, and Inception have achieved state-of-the-art performance in plant disease classification, they are computationally expensive, data-hungry, and often lack interpretability. In contrast, traditional machine learning models are lightweight and explainable but struggle with high-dimensional image data. Very few studies have attempted to bridge these two paradigms by combining deep feature extraction with dimensionality reduction and hybrid classifiers. To address this gap, our study employs ResNet to extract rich feature representations from plant leaf images, applies Principal Component Analysis (PCA) to reduce dimensionality and computational overhead, and then leverages both machine learning and deep neural network classifiers for final prediction. This unique ResNet-PCA + ML/DNN framework offers a balance between efficiency and accuracy, enabling high-performance classification without the full cost of end-to-end CNN training, thereby making the approach more practical for real-world agricultural applications where computational resources and annotated data may be limited.

- The study underscores the critical role of early disease identification in agriculture and proposes a hybrid modeling pipeline that addresses the limitations of conventional expert-based and purely DL approaches.
- This research systematically explores hybrid combinations, including LR+DNN, RF+DNN, GB+DNN, KNN+DNN, and XGB+DNN. The experimental results demonstrated competitive performance across all hybrids, with accuracies of 91.78% for RF+DNN, 93.78% for XGB+DNN, 90.22% for GB+DNN, 80.89% for KNN+DNN, and 96.22% for LR+DNN.
- Through detailed analysis of classification metrics and learning curves, it was established that the LR+DNN hybrid consistently outperformed all other models, achieving the highest accuracy of 96.22%. This finding validates the effectiveness of hybrid learning strategies in plant disease recognition and sets a new benchmark for future research in agricultural AI applications.

This paper is organized into six sections. Section 2 reviews previous work related to the subject matter. In Section 3, we describe the dataset and system design, followed by Section 4, which outlines the proposed approach utilized to achieve the results. The findings are detailed in Section 5. Finally, Section 6 concludes the paper and discusses potential future directions. Additionally, **Table 1** lists the acronyms used throughout this paper.

**Table 1:** List of acronyms.

Full form	Acronyms
Machine Learning	ML
Deep Learning	DL
Principal Component Analysis	PCA
Random Forest	RF
Extreme Gradient Boosting	XGB
Gradient Boosting	GB
K Nearest Neighbors	KNN
Logistic Regression	LR
Deep Neural Network	DNN
Local Interpretable Model-Agnostic Explanation	LIME
Receiver Operating Characteristic	ROC

## 2 Literature review

Authors in (Reddy et al., 2024) presented a DNN-based model to detect plant diseases in leaves by using their pictures and trained on the New Plant Diseases (Augmented) data that contains information about 38 classes, and the Rice Leaf dataset, which is associated with 4 classes. Different features, including grey level and shape features of leaves, are extracted by the model to analyze the characteristics of leaves comprehensively. These features are total area, infected area, perimeter, coordinate of centroid, mean intensity, entropy, eccentricity, energy, homogeneity, and dissimilarity. The hyperparameters considered included epoch, batch size, type of activation, and dropout rates, which resulted in the model achieving accuracy between 96 and 99 percent, outperforming traditional ML models. In (Chaitanya and Posonia, 2024), the author suggests the development of an efficient plant disease recognition system that would operate in a mobile format. The framework enhances the visual representation of plants by extracting two sets of features: one using CO-KMC segmentation and the MRV-BWO network to capture color, shape, and texture features, and another using the Multi-scale Dilated Attention CNN (MSDA-CNN) network to capture deep semantic features. These are fused with a weighted strategy referred to as MRV-BWO and then classified using a Hybridized DNN-RNN model, which is further optimized with MRV-BWO. Findings reveal that the method is effective in plant disease detection because it offers better resources and performance compared to conventional methods.

In (Jafar et al., 2024), the authors reviewed the use of artificial intelligence in revolutionizing agricultural processes by considering the approach of AI and IoT in detecting plant diseases used in tomato, chili, potato, and cucumber crops. It explains the disease detection process in terms of the significant stages of image acquisition, preprocessing, segmentation, feature selection, and classification, comparing different ML and DL methods applied in recent research. The study also notes that often-utilized datasets are used to evaluate the most frequent diseases, outlining their symptoms and pinpointing the drawbacks of existing detection methods. Besides, it focuses on future opportunities of combining AI with IoT technologies, e.g., smart drones to monitor plants in the field, which provides information about the actual challenges and possibilities of developing automated disease diagnosis of plants. Authors in (Dey et al., 2024) presented a DL-based system for automated plant disease detection, integrating precision agriculture and transfer learning via pre-trained CNN models (AlexNet, VGG16, and VGG19). The study utilized the PlantVillage dataset, which comprises healthy and diseased leaves, and preprocessed the images to enhance their quality. The models were then trained on 80 percent of the data and validated on 20 percent. These findings revealed that all models had high performance in the classification of plant diseases, and AlexNet had the highest performance, which indicated the possibilities of DL as a viable mechanism of early disease detection and proper management of crops. Shahi et al (Shahi et al., 2023). presented a review of the latest advances in UAV-based remote sensing for detecting crop diseases, with a particular emphasis on combining sophisticated sensors, image processing methods, and DL applications. It also introduces a taxonomy to conveniently sort the available literature as well as comparing the performance of ML and DL approaches to estimate the disease, and underlining the importance of using UAV imagery to increase the accuracy. The study concludes by presenting current challenges, opportunities, and recommendations for future research, highlighting the use of UAVs as a potent means of detecting early diseases and precision agriculture.

Authors in (Raman and Jayaraman, 2025) presented a hybrid deep learning model by Dual Branch Convolutional Graph Attention Neural Network (DB-CGANNet) to detect rice leaf disease. Noise reduction is done by Upgraded Weighted Median Filtering (Up-WMF), and AG-CLAHE does contrast enhancement. The Discrete Wavelet Transform (DWT), Gray Level Run Length Matrix (GLRLM), and VGG19 are used to extract features, and the Bio-Inspired Artificial Hummingbird (BI-AHB) algorithm is optimal in the selection of features. The dual-branch model is an extension of handcrafted and deep features, achieving an accuracy of 98.9% in the Rice Leaf Diseases Dataset and 99.08% in the Rice Disease Images Dataset, which is higher than the known methods and is beneficial to the sustainable farming of rice. In (Haridasan et al., 2023), the authors suggested a deep learning-based automated system to detect and classify paddy plant diseases to enhance crop health and productivity (ID). The system combines computer vision technology with machine learning and deep learning to decrease the reliance on traditional diagnostic approaches. Segmentation is then used to separate diseased areas after preprocessing of the image, and is followed by five major rice diseases common in the Indian fields. To achieve an accurate classification, a hybrid model that integrates the support vector machines (SVM) with convolutional neural networks (CNN) is used. The system was able to reach a validation accuracy of 91.45 percent and goes ahead to offer predictive remedies to assist farmers and agricultural organizations.

The systematic literature review (SLR) on plant disease detection proposed by (Shafik et al., 2023) consists of the details of the motivations, approaches to classification, data, and difficulties, as well as perspectives. The researchers scoured through 1349 articles in the leading databases. They settled on 176 studies examining applications of AI, ML, and DL in the agricultural context, especially vision- and hyperspectral-based approaches to grapes, rice, apples, maize, and other crops. They note that SVM and LR models perform better than the conventional classifiers and that more recent developments follow on the path of CNNs with attention and transfer learning. The paper also surveys 11 datasets (9 publicly available) and observes limitations, including dataset size, the absence of standard metrics of evaluation, and constraints in localizing the disease. Finally, the paper stipulates that to substantiate the proposed model, it will be necessary to have lightweight, robust models that can be implemented on small devices and are scalable to a variety of crops and diseases. Authors in (Kulkarni and Shastri, 2024) suggested an automatic system based on convolutional neural networks (CNN) to detect and classify rice leaf diseases to assist in the timely diagnosis and treatment of the disease in agriculture. The model, utilizing the concept of image processing, is designed to handle challenging scenarios such as updating background and background illumination, which would have been problematic for traditional manual identification methods. The CNN is well-portrayed as it can correctly classify rice leaf images sampled across various environments, which shows excellent robustness in the agricultural environment. As a result of this method, the authors attained a setting accuracy of 95 percent, indicating the potential of DL solutions in precision farming and creating an effective, automated diagnostic tool for farmers to identify diseases.

## 3 Dataset and system design

Experiments in this paper rely on the open resource of plant leaf images known as PlantVillage, currently under development and growing in popularity, and which has been carefully curated to assist in research on plant disease detection (Mohanty et al., 2016). The dataset is available on GitHub and Kaggle and contains thousands of pictures of different types of crops, such as tomatoes, potatoes, and bell peppers, and a disease type or health outcome accompanies the category. Examples of PlantVillage were taken in controlled light and on a uniform ground, which increases the consistency and lessens noise, and consequently, the feature learning of both classical and DL networks.

The original collection of images in PlantVillage consists of around 54,306 images of 14 crop species and 26 disease conditions (including healthy), with 38 unique classes of crop-disease from which we have selected the 15 unique classes to work that are shown in **Table 2**. The dataset was modeled in controlled settings using uniform backgrounds, similar light, and mostly individual leaves photographed in the flat position in order to reduce visual noise and environmental differences. To facilitate the needs of various researchers, there are various versions of the images: the original RGB (color) versions, a grayscale version and a segmented version in which the background is eliminated and the color correction is used to minimize possible bias due to lighting or background effects. To ensure consistency in the studies, all pictures are downsampled to 256 × 256 pixels before the training of models.

**Table 2:** Class distribution of plant disease dataset.

Class	Number of images
Pepper_bell_Bacterial_spot	997
Pepper_bell_healthy	1478
Potato_Early_blight	1000
Potato_Late_blight	1000
Potato_healthy	152
Tomato_Bacterial_spot	2127
Tomato_Early_blight	1000
Tomato_Late_blight	1909
Tomato_Leaf_Mold	952
Tomato_Septoria_leaf_spot	1771
Tomato_Spider_mites_Two_spotted_spide_mite	1676
Tomato_Target_Spot	1404
Tomato_Tomato_YellowLeaf_Curl_Virus	3208
Tomato_Tomato_mosaic_virus	373
Tomato_healthy	1591

The data includes a broader set of disease states, and healthy leaves are labeled, which allows the multi-class classification with fine-grained accuracy. In this paper, a subset of the dataset of the PlantVillag, including crop-disease pairs, including those affecting pepper, potato and tomato, was used to test the performance of the hybrid models by the ML+DNN designs. In order to have a strong stratified sampling, the dataset was separated into training, validation and test batches, with similar proportions of classes in those subsets. This method resulted in a high degree of strength of the evaluation measures in measuring the generalization potential of the models in different disease categories, and even in rare classes.

### 3.1 Data preprocessing

A classification system performs well based on the caliber of the feature representation of the system. About plant disease detection, the leaf images usually show some changes in color, texture, and morphology, all of which can be considered as possible disease severity predictors. Since these aspects were needed in detail, the two-stage preprocessing pipeline was used. At the first step, a handcrafted feature was obtained according to color, texture, and shape descriptors. The second stage also entailed the acquisition of deep convolutional neural representations via a pretrained ResNet50 model. Lastly, feature fusion was used to merge both representations to give a more discriminating feature space. At the handcrafted feature extraction step, all images were uniformly reduced in size to 224 × 224 pixels. The initial descriptors were related to color information, which is vital in distinguishing diseased leaf occurrences from healthy ones, as most infections have a noticeable discoloration. The color histograms were formed in HSV spaces, as well as in LAB spaces, to capture the changes in chroma and be resistant enough to differences in illumination. The channel *c* is mathematically described in **Equation 1**, where the histogram of a channel is given by the bin numbered by *b*, and the intensity of the *i*-th pixel is written as $I_{c}\left(i\right)$, and δ(·) is its indicator.

(1) $$H_{c}\left(b\right)=\sum_{i=1}^{N}\delta \left(I_{c}\left(i\right)\in b\right).$$

To derive even more indicative color hues, the predominant color was calculated through *k*-means clustering in three classes, i.e., *k* = 3. The mean of every cluster was computed as given in **Equation 2** where n_j represents the number of pixels under cluster j.

(2) $$C^{*}=\frac{1}{n_{j}}\sum_{i=1}^{n_{j}}x_{i},\;\;\;x_{i}\in {\mathbb{R}}^{3}.$$

There were also texture descriptors used that quantify the pathogen-induced micro-patterns on the leaf surface. Local Binary Patterns (LBP) were calculated by **Equation 3** where $g_{c}$ refers to the gray intensity of the center pixel, $g_{p}$ corresponds to the neighbor under consideration, and the thresholding function is denoted by *s*(*z*).

(3) $$LBP_{P,R}\left(x,y\right)=\sum_{p=0}^{P-1}s\left(g_{p}-g_{c}\right)\;2^{p},\;s\left(z\right)=\{\begin{array}{ll} 1, & z\geq 0\\ 0, & z<0 \end{array}$$

Besides LBP, statistical texture features, such as Gray-Level Co-occurrence Matrices (GLCM), were also underlying. Contrast and Angular Second Moment (ASM) were calculated, respectively, as indicated below in **Equations 4**, **5**, where the variables P(i,j) give the normalized probability of the co-occurrence of gray levels i and j.

(4) $$\text{Contrast}=\sum_{i,j}{\left(i-j\right)}^{2}P\left(i,j\right),$$

(5) $$\text{ASM}=\sum_{i,j}P{\left(i,j\right)}^{2}.$$

Shape descriptors were extracted to pick up irregularities in the structure of diseased leaves. The three morphological features that were kept included leaf area (*A*), perimeter (*P*) and circularity (*C*) after segmentation by using contour detection. Circularity is also very applicable in that damage inflicted on the leaves can alter the shape of the leaf. It is given as in **Equation 6**.

(6) $$C=\frac{4\pi A}{P^{2}}.$$

Each handcrafted descriptor was concatenated to form a single feature vector. The number of classes was then selected to ensure equal numbers in each category, achieved by randomly selecting 150 images from each category. Although handcrafted features offer expressive properties that present perceptible descriptors in terms of color, texture, and shape, in complex semantics, these features are unable to capture the semantics. To counter this, deep feature representations through a ResNet50 network pretrained in ImageNet were used. The network was cut after the global average pooling (GAP) layer, and its output served as a compact, but highly expressive descriptor. Formally, the deep feature vector can be defined as in Equation 7, i.e., the transform *ϕ*(*x*;*θ*) of the input *x* is a conformally-learned convolutional operation with parameters *θ*, which are pretrained on a separate task.

(7) $$f_{deep}=\text{GAP}\left(\phi \left(x;\theta \right)\right).$$

Lastly, to maximize the advantages of the two forms of representations, namely, handcrafted and deep representations, they were fused by concatenating the two vectors. The fused representation is written out in Equation 8.

(8) $$F=\left[f_{traditional}\;\|\;f_{deep}\right],$$

Where “traditional” refers to handcrafted descriptors, and “deep” refers to CNN features. PCA (PCA) was next used to reduce dimensionality by retaining the most informative components. To visually verify the effectiveness of the technique and to demonstrate the capacity for distinguishing between the classes of diseases, t-SNE was utilized in projecting the features into two dimensions. Moreover, RF importance scores demonstrated the discriminative value of handcrafted features at a low level as well as deep features at a high level. Such a combined preprocessing pipeline, therefore, generated sufficiently balanced, high-dimensional and semantically rich feature vectors that formed the basis of the hybrid ML+DNN classification framework proposed here.

### 3.2 High level system design

**Figure 1:**
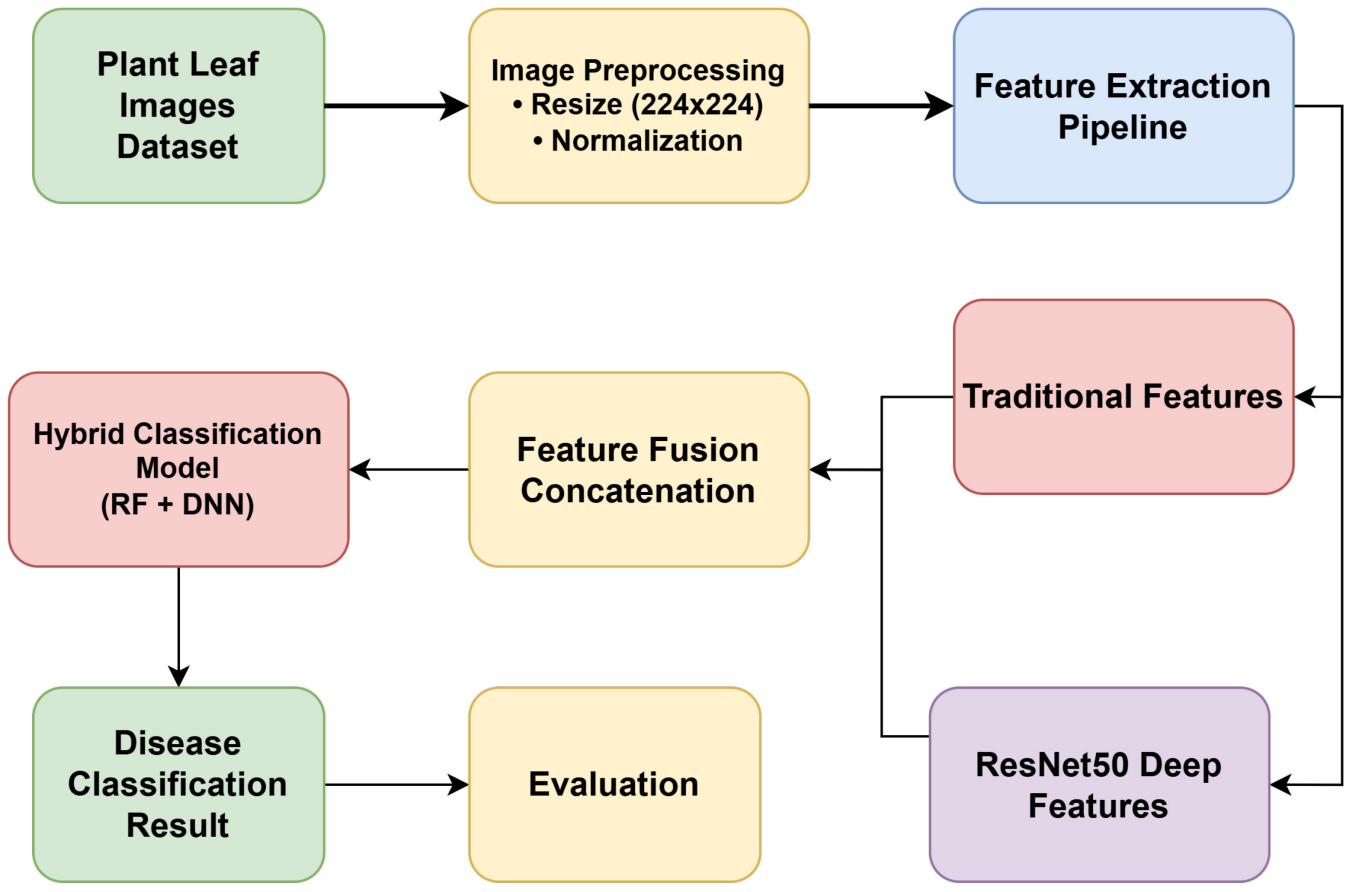
Basic system architecture for plant disease detection.

The system proposed for plant disease detection is a hybrid architecture that integrates conventional image-processing routines with deep representation approaches to achieve robust classification, as illustrated in **Figure 1**. It starts with the gathering of plant leaf photographs, which is marked as the core dataset. These images undergo processing, including resizing to a fixed resolution (224 x 224) and normalization, to ensure uniformity and improve model performance. A feature extraction pipeline processes the data within the scope of the problem after preprocessing; two parallel methods are used. The first one involves extracting conventional features, including color histograms, dominant color values, Local Binary Patterns (LBP), Gray-Level Co-occurrence Matrix (GLCM) features, and shape descriptors. The second method uses the pre-trained convolutional neural networks (e.g., the ResNet50, VGG16, and EfficientNetB0) to extract deep representations. The feature fusion takes place based on the concatenation of the results of these two streams, thus forming a highly detailed feature set. The fused representation is then sent on to a hybrid classification model, which is a hybrid of RF and DNN classifiers, to maximize predictive accuracy and generalization. The system then provides the classification result, indicating whether the input leaf falls into a diseased or healthy type. Such a hybrid design forms higher reliability through integration of the strengths of handcrafted features and DL-based features to detect disease in plants accurately.

## 4 Proposed approach

**Figure 2:**
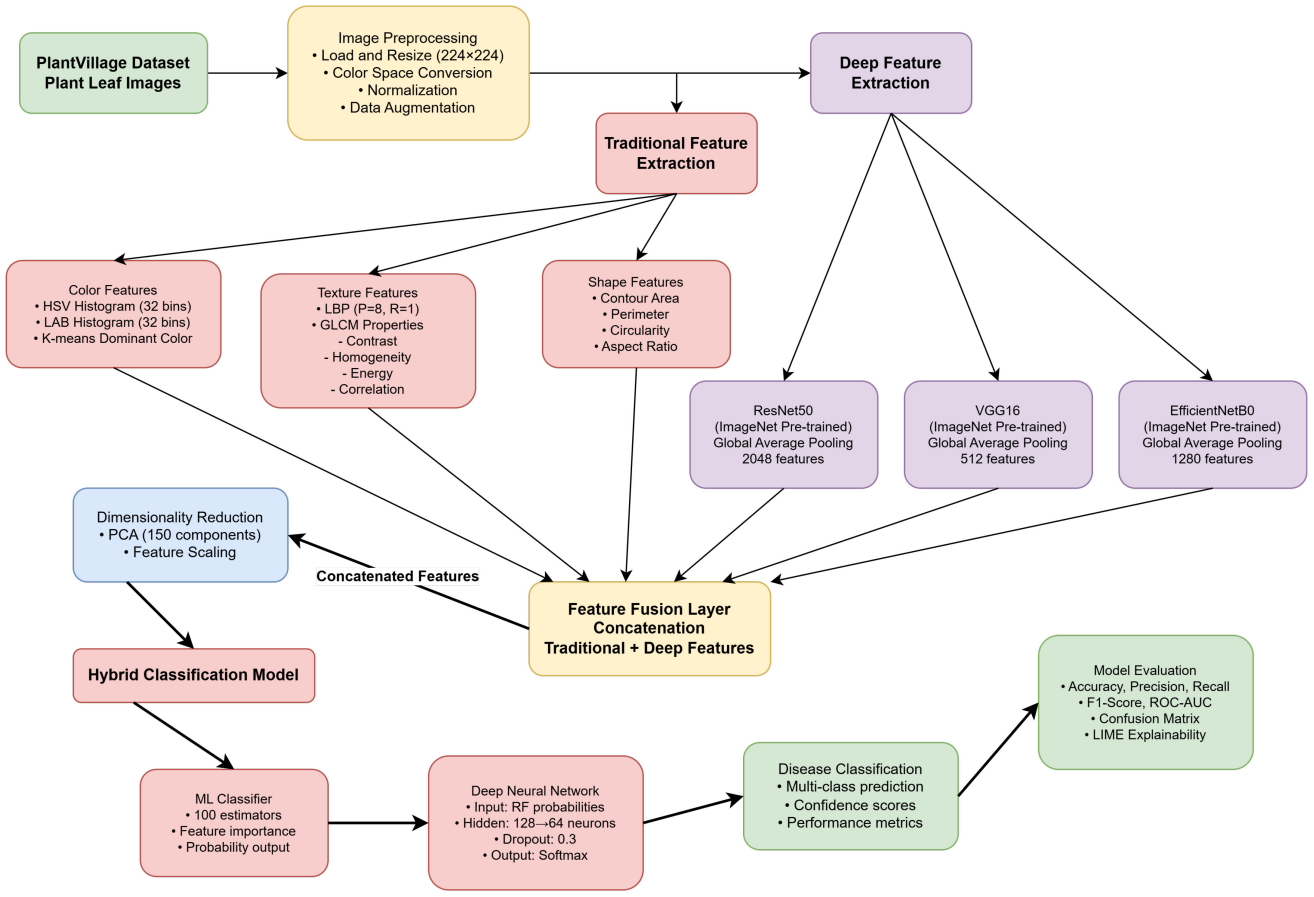
Detailed framework of the proposed hybrid ML+DL model.

The proposed approach presents the Hybrid-Plant-Disease-Detection-Framework, which is an efficient combination of image processing and deep learning to support reliable detection of plant-leaf diseases. As given in **Figure 2** and given in **Algorithm 1**, the framework uses a sequential pipeline that starts with the image preprocessing, feature-extraction, feature-fusion, dimensionality reduction and the final classification step. The combination of handcrafted radiomic features and deep features based on CNNs allows the approach to capture textural features at low levels and semantic patterns at high levels, coupled with the ability to improve accuracy and generalization across a range of disease classes due to the application of ensemble-based classification.

Algorithm 1

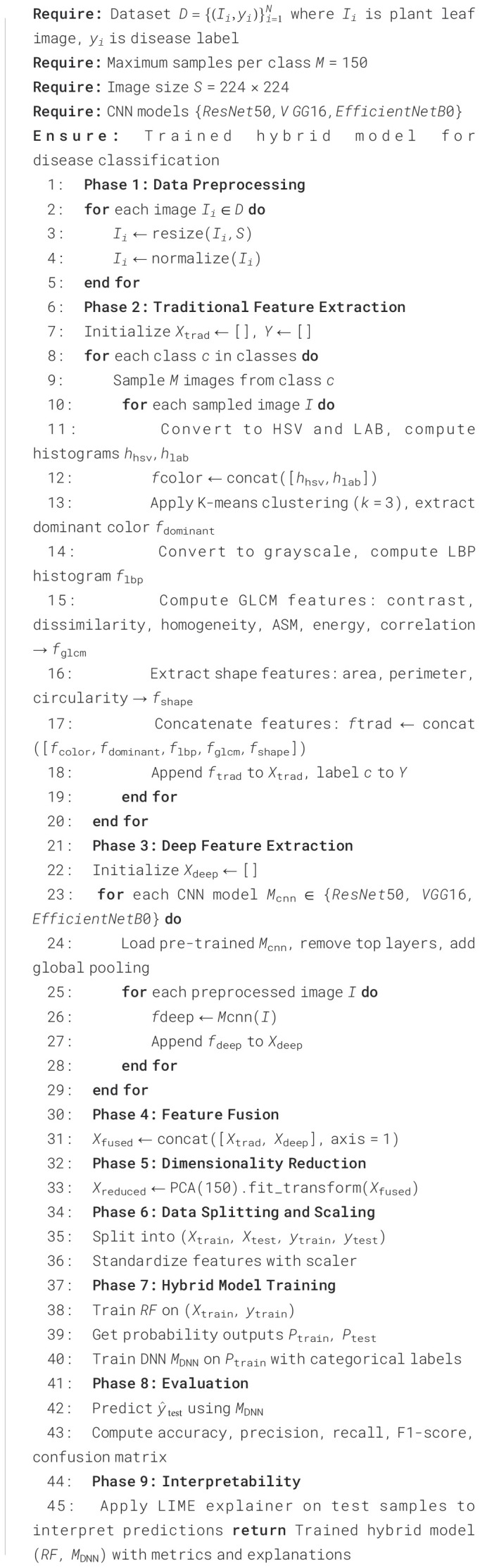

### 4.1 Machine learning models

The work applies a combination of the different classic ML classifiers as sublearners in the hybrid ML-DNN architecture. The features, reduced using principal component extraction and passed through the ResNet model, are used with each classifier to achieve initial predictions, which are subsequently refined using a DNN. The range of selected algorithms (RF, XGB, GB, KNN, LR) was chosen owing to their advantageous combination of properties in terms of non-linearity of decision boundaries, noise robustness, and generalization to a wide variety of plant disease types.

#### 4.1.1 Random forest

RF is an ensemble ML algorithm that trains many decision trees. In the case of classification, the mode of predictions is output (Salman et al., 2024). The fact is that PCA transforms the features, making them one-dimensional and reducing the dimensionality of the features, which in turn reduces overfitting and computational complexity in this work. RF exhibits high noise and variability robustness in plant disease images due to the use of bootstrap aggregation (bagging) and feature randomness, making it applicable to more complex visual patterns in the dataset. The decision rule takes the form of **Equation 9**.

(9) $$\hat{y}=\text{mode}{\left\{h_{t}\left(x\right)\right\}}_{t=1}^{T}$$

where *h_t_*(**x**) denotes the prediction from the *t*-th decision tree, and *T* is the total number of trees.

#### 4.1.2 Extreme gradient boosting

XGBoost is a fast, computationally efficient, and optimization-friendly extension of the GB framework that supports regularization, parallel acceptance, and efficient tree pruning to enhance predictive performance (Wen et al., 2023). Here, given ResNet PC features, resistance to noise and accurate continuation of complex classes are carried out by the XGBoost of this framework. Overfitting is reduced through the built-in L1 and L2 regularization terms, and the scalability allows fast training even in agents with high-dimensional collected features. It will be presented by Equation 10.

(10) $${\hat{y}}_{i}=\sum_{k=1}^{K}f_{k}\left(x_{i}\right),\;f_{k}\in ℱ$$

where ℱ represents the space of regression trees and K is the number of trees. The overall objective combines the loss function and regularization as shown in Equation 11.

(11) $$\text{Obj}=\sum_{i=1}^{n}L\left(y_{i},{\hat{y}}_{i}\right)+\sum_{k=1}^{K}\text{Ω}\left(f_{k}\right)$$

#### 4.1.3 Gradient boosting

GB constructs additive models through ML by sequentially fitting a decision tree to the error of each previous decision tree, fitting errors in an additive manner (Zhang et al., 2024). Hence, the model becomes more accurate as it proceeds. GB is also good at finding subtle variability in feature patterns, which in the case of visually similar classes of plant diseases becomes critical as to how to tell them apart. During combination with reduced deep features by PCA, GB provides a good bias-variance tradeoff and enhances the overall hybrid framework in terms of discriminative power. In Equation 12, the additive update rule is described.

(12) $$F_{m}\left(x\right)=F_{m-1}\left(x\right)+\gamma_{m}h_{m}\left(x\right)$$

where $F_{m}$ is the ensemble at iteration m, $h_{m}$ is the weak learner, and $\gamma_{m}$ is the step size.

#### 4.1.4 K-Nearest neighbors

KNN is a non-parametric learning algorithm applied in instance-based learning to classify a new sample based on the majority of the samples among its KNN in the feature space (Halder et al., 2024). PCA transformation enhances the efficiency of KNN, as it reduces the cost associated with the distance metric by minimizing noise and feature dimensionality. KNN is a baseline nonlinear classifier in this paradigm, which is highly interpretable and utilizes the abundant semantic representation afforded by ResNet. This is written as Equation 13.

(13) $$\hat{y}=\text{mode}\left\{y_{i}\;|\;x_{i}\in N_{k}\left(x\right)\right\}$$

where $N_{k}\left(x\right)$ denotes the set of k nearest neighbors of x.

#### 4.1.5 Logistic regression

The LR is one of the linear classification algorithms that estimates the probability of a class based on a logistic sigmoidal function (Zaidi and Al Luhayb, 2023). Although it is a simple method, LR can act as a useful scale of effectiveness of deep feature extraction and PCA transformation. Using LR on the compressed ResNet representations, the model embodies linear separable patterns within the dataset and can be used as an effective yet computationally efficient node in the hybrid ML-DNN pipeline. The probability in each class will be binary as shown in Equation 14.

(14) $$P(y=1|x)=\sigma \left(w^{⊤}x+b\right)=\frac{1}{1+e^{-\left(w^{⊤}x+b\right)}}$$

where w is the weight vector, b is the bias, and σ(·) is the sigmoid activation.

### 4.2 Dense neural network

The proposed structure has two stages with an ML classifier paired with a specific DNN dedicated to each of them (Girdhar et al., 2023). The first step entails training the ML model of interests on the PCA-reduced ResNet features to produce intermediary predictions or probability scores. Mathematically, it corresponds to Equation 15, where $p_{j}$ refers to the output of the ML model number j.

(15) $$p_{j}=M_{j}\left(X_{PCA}\right)$$

Here, $X_{PCA}\in {\mathbb{R}}^{n\times k}$ represents the k-dimensional features obtained after PCA, and $p_{j}\in {\mathbb{R}}^{n\times m}$ is the predicted probability distribution over m classes.

In stage two, the output $p_{j}$ is the input to the relevant DNN, which further non-linearly transforms the data. In the forward pass, the computation at a particular hidden layer l is given as the formula Equation 16.

(16) $$z^{\left(l\right)}=W^{\left(l\right)}a^{\left(l-1\right)}+b^{\left(l\right)}$$

The activation for layer l is then obtained by applying a nonlinear activation function g(·), such as ReLU, as shown in Equation 17.

(17) $$a^{\left(l\right)}=g\left(z^{\left(l\right)}\right)$$

Last, a softmax activation is applied on the final layer of the DNN to yield the optimized score over *m* disease classes of plant diseases, which is shown in Equation 18.

(18) $${\hat{y}}_{j}=\frac{e^{z_{j}}}{\sum_{c=1}^{m}e^{z_{c}}}$$

The Dense Neural Network architecture applied in the given work is represented in **Table 3**. It comprises 512 neurons in the input layer, activated by the Rectified Linear Unit (ReLU) form, with subsequent alternating dense and dropout layers to enhance generalization and to avert overfitting. Particularly, dropout layers at a rate of 0.3 are used between each dense layer in the hidden layer and a random amount of neurons are deactivated during training to minimize co-adaptation of weights. The network sequentially decreases dimensionality with the first hidden layer having 256 neurons, the second layer having 128 neurons, then arriving at the output layer that has final units neurons and final activation, which performs an activation suitable to the classification task (softmax on multi-class or sigmoid on binary classes). This structure strikes a balance between representation power and regularization, which helps optimize the outputs of each ML model.

**Table 3:** Dense neural network (DNN) architecture used in the proposed framework.

Layer no.	Layer type	Units	Activation/Dropout
1	Dense (Input)	512	ReLU
2	Dropout	–	0.3
3	Dense	256	ReLU
4	Dropout	–	0.3
5	Dense	128	ReLU
6	Dropout	–	0.3
7	Dense (Output)	15	softmax

### 4.3 Principal component analysis

PCA is a dimension reduction method that projects information onto a smaller subspace while maximizing the retained variance. On the same note, PCA is used with the feature vectors of the already-trained ResNet model in this research to minimize redundancy and computational complexities, hence enhancing the efficiency of training the model. Given a dataset $\;X\in {\mathbb{R}}^{n\times d}$, the first step is to center the data by subtracting the mean from each feature. The covariance matrix C is then computed as shown in Equation 19.

(19) $$C=\frac{1}{n-1}X^{⊤}X$$

Eigen decomposition shown in Equation 20 is performed on **C** to obtain eigenvalues $\lambda_{i}$ and eigenvectors $v_{i}$.

(20) $$Cv_{i}={\text{λ}}_{i}v_{i}$$

The selection is then made based on the largest eigenvalues, using the k top eigenvectors to constitute the projection matrix: $W_{k}$. We also have a reduced representation of the set of features, denoted as Z, which is calculated using Equation 21.

(21) $$Z=XW_{k}$$

### 4.4 Model explainability using LIME

This study uses the technique of LIME to achieve *post-hoc* interpretability of the hybrid ML+DNN models. LIME uses locally faithful explanations constructed by approximating the interpretable surrogate model to the complex one in the area of a prediction. LIME takes a black-box classifier in the form of a classifier *f*, and an instance *x*, and uses perturbed samples surrounding *x* whose respective predicted output by *f* is returned by LIME. Each sample (or point) *z* is assigned with the locality-awareweight $\pi_{x}\left(z\right)$ which favors or prioritizes points closer to x as in Equation 22.

(22) $$\pi_{x}\left(z\right)=\exp\;\left(-\frac{D{\left(x,z\right)}^{2}}{\sigma^{2}}\right)$$

with a distance metric D(·) and the kernel width σ. Then, a simple, interpretable model g∈G (e.g., sparse linear model) is learned that minimizes the objective function as indicated in Equation 23.

(23) $$\xi \left(x\right)=\;\underset{g\in G}{\text{arg }\min}ℒ\left(f,g,\pi_{x}\right)+\text{Ω}\left(g\right)\;$$

where ℒ measures the fidelity of g to f in the locality defined by $\pi_{x}$, and Ω(g) enforces model simplicity.

## 5 Results, discussion and limitations

The presentation of the experimental assessment of the proposed hybrid ML-DNN framework is carried out in this section. The experiments aimed to evaluate the classification accuracy of various ML models, incorporating Dense Neural Networks (DNNs) with ResNet-extracted and PCA-reduced features. The assessment is based on classical performance metrics, e.g., accuracy and F1-score, and interpretability metrics, including the LIME. Standard metrics and visual diagnostics, including prediction training and validation curves, confusion matrices, and ROC curves, are used to report the results and are supplementary to providing a view on robustness, reliability, and explainability of the proposed framework.

Along with these scalar metrics, training and validation accuracy/loss should be used to analyze the model convergence behavior, which is helpful to correct some problems like overfitting or underfitting. Stable generalization implies a constant decrease in the difference between training and validation performance per epoch. Additionally, the confusion matrix gives a precise perspective of the legitimacy of the classification performance in each class. The Confusion Matrix demonstrates more insight than aggregate metrics because, by plotting actual vs. predicted labels, the user should also be able to see which types of misclassifications a model is prone to (as opposed to the effective average result). The ROC curve is also depicted to assess the ratio between the actual positive rate (TPR) and the false positive rate (FPR) at different thresholds. The ROC-based Area-Under-the-Curve (AUC) would be a good measure of the discriminative ability of the classifier. Lastly, the interpretability of the models is guaranteed through the application of LIME. LIME only outputs local surrogates to complex hybrid ML-DNNs, where the most essential features for making predictions are emphasized. This makes the model trustworthy as it provides transparency in decision-making, which is crucial in key agricultural applications, particularly in detecting plant diseases.

### 5.1 Results

Each of the proposed hybrid models was tested using classification reports concerning each of the classes of crop diseases. **Tables 4**-**8** show the precision, recall and F1-score values in all disease categories under a variety of model combinations, and **Table 9** gives the general performance of each model. The RF+DNN model presented in **Table 4** achieved a competitive accuracy of 91.7%, with high precision and recall values across nearly all classes. Indicatively, Tomato mosaic virus and Tomato healthy achieved the same F1-score of 0.98, and courses like Tomato Early blight (F1 = 0.79) and Tomato Spider mites (F1 = 0.86) were more problematic, thus could be compared as hard to distinguish virus diseases which may be visually related.

**Table 4:** Classification report for RF+DNN.

Class	Precision	Recall	F1-score
Pepper_bell_Bacterial_spot	1.00	0.93	0.97
Pepper_bell_healthy	0.94	1.00	0.97
Potato_Early_blight	0.93	0.87	0.90
Potato_Late_blight	0.93	0.90	0.92
Potato_healthy	0.91	1.00	0.95
Tomato_Bacterial_spot	0.90	0.93	0.92
Tomato_Early_blight	0.85	0.73	0.79
Tomato_Late_blight	0.96	0.83	0.89
Tomato_Leaf_Mold	0.88	0.97	0.92
Tomato_Septoria_leaf_spot	0.96	0.87	0.91
Tomato_Spider_mites_Two_spotted_spider_mite	0.82	0.90	0.86
Tomato_Target_Spot	1.00	0.83	0.91
Tomato_Tomato_YellowLeaf_Curl_Virus	0.81	1.00	0.90
Tomato_Tomato_mosaic_virus	0.97	1.00	0.98
Tomato_healthy	0.97	1.00	0.98
Weighted Avg	0.92	0.92	0.92

The generalization performance of the XGB+DNN model was better in terms of overall accuracy of 93.8% observed in **Table 5**. It is noteworthy that it got perfect classification concerning *Tomato_mosaic_virus* (F1 = 1.0) and an extremely high score with *Potato_healthy* and *Tomato_YellowLeafCurlVirus*. Nevertheless, the *Tomato_Early_blight* turned out to be a problematic group, too, as its precision was relatively low (0.66), but its recall was quite high (0.97), indicating false-positive prone.

**Table 5:** Classification report for XGB+DNN.

Class	Precision	Recall	F1-score
Pepper_bell_Bacterial_spot	1.00	0.93	0.97
Pepper_bell_healthy	0.97	1.00	0.98
Potato_Early_blight	0.97	0.93	0.95
Potato_Late_blight	0.97	0.93	0.95
Potato_healthy	0.97	1.00	0.98
Tomato_Bacterial_spot	1.00	0.87	0.93
Tomato_Early_blight	0.66	0.97	0.78
Tomato_Late_blight	0.89	0.80	0.84
Tomato_Leaf_Mold	0.93	0.93	0.93
Tomato_Septoria_leaf_spot	0.93	0.87	0.90
Tomato_Spider_mites_Two_spotted_spider_mite	1.00	0.93	0.97
Tomato_Target_Spot	0.97	0.97	0.97
Tomato_Tomato_YellowLeaf_Curl_Virus	1.00	0.97	0.98
Tomato_Tomato_mosaic_virus	1.00	1.00	1.00
Tomato_healthy	0.97	0.97	0.97
Weighted Avg	0.95	0.94	0.94

Contrarily, the overall accuracy of the GB+DNN model was lower by 0.2% as indicated in **Table 6**. Whereas the model achieved outstanding performance on various classes, such as *Pepper*_*healthy* and *Tomato*_*Bacterial*_*spot*, it performed poorly on other courses, like *Tomato*_*Late*_*blight*, with recall decreasing to 0.69, thus increasing its vulnerability compared to RF+DNN and XGB+DNN.

**Table 6:** Classification report for GB+DNN.

Class	Precision	Recall	F1-score
Pepper_bell_Bacterial_spot	0.96	0.87	0.91
Pepper_bell_healthy	1.00	0.93	0.97
Potato_Early_blight	0.93	0.93	0.93
Potato_Late_blight	0.93	0.83	0.88
Potato_healthy	0.94	0.97	0.95
Tomato_Bacterial_spot	0.97	0.93	0.95
Tomato_Early_blight	0.84	0.87	0.85
Tomato_Late_blight	0.69	0.90	0.78
Tomato_Leaf_Mold	0.93	0.87	0.90
Tomato_Septoria_leaf_spot	0.77	0.90	0.83
Tomato_Spider_mites_Two_spotted_spider_mite	0.88	0.93	0.90
Tomato_Target_Spot	0.93	0.87	0.90
Tomato_Tomato_YellowLeaf_Curl_Virus	0.96	0.87	0.91
Tomato_Tomato_mosaic_virus	0.97	0.93	0.95
Tomato_healthy	0.97	0.93	0.95
Weighted Avg	0.91	0.90	0.90

Among all the hybrid models included in **Table 7**, the KNN+DNN model got the lowest performance with an overall accuracy of 80.9%. There were a few bad classes, such as the class *Tomato*_*Late*_*blight* (F1 = 0.63) and the class *Tomato*_*Target*_*Spot* (F1 = 0.70). This indicates the low compatibility of KNN with deep features, as it is sensitive to high-dimensional feature spaces.

**Table 7:** Classification report for KNN+DNN.

Class	Precision	Recall	F1-score
Pepper_bell_Bacterial_spot	0.80	0.80	0.80
Pepper_bell_healthy	0.93	0.83	0.88
Potato_Early_blight	1.00	0.83	0.91
Potato_Late_blight	0.78	0.83	0.81
Potato_healthy	0.94	1.00	0.97
Tomato_Bacterial_spot	0.97	0.97	0.97
Tomato_Early_blight	0.70	0.63	0.67
Tomato_Late_blight	0.71	0.57	0.63
Tomato_Leaf_Mold	0.78	0.83	0.81
Tomato_Septoria_leaf_spot	0.70	0.77	0.73
Tomato_Spider_mites_Two_spotted_spider_mite	0.70	0.63	0.67
Tomato_Target_Spot	0.62	0.80	0.70
Tomato_Tomato_YellowLeaf_Curl_Virus	0.87	0.87	0.87
Tomato_Tomato_mosaic_virus	0.91	0.97	0.94
Tomato_healthy	0.80	0.80	0.80
Weighted Avg	0.81	0.81	0.81

Among all of the models, LR+DNN displayed in **Table 8** had the highest accuracy of 96.2%, the best performing model. It gave almost perfect classification in the vast majority of the classes, with the exceptions of *Potato*_*healthy*, *Tomato*_*Bacterial*_*spot*, and *Tomato*_*Target*_*Spot* that reported only slightly lower (but still nearly 1.0) F1-scores. The model performed well even in the case of problematic classes like *Tomato*_*Early*_*blight* (F1 = 0.92).

**Table 8:** Classification report for LR+DNN.

Class	Precision	Recall	F1-score
Pepper_bell_Bacterial_spot	0.97	0.93	0.95
Pepper_bell_healthy	0.97	0.97	0.97
Potato_Early_blight	0.97	1.00	0.98
Potato_Late_blight	1.00	0.97	0.98
Potato_healthy	1.00	1.00	1.00
Tomato_Bacterial_spot	1.00	1.00	1.00
Tomato_Early_blight	0.90	0.93	0.92
Tomato_Late_blight	0.93	0.87	0.90
Tomato_Leaf_Mold	0.94	0.97	0.95
Tomato_Septoria_leaf_spot	0.93	0.93	0.93
Tomato_Spider mites_Two_spotted_spider_mite	0.93	0.93	0.93
Tomato_Target_Spot	1.00	0.97	0.98
Tomato_Tomato_YellowLeaf_Curl_Virus	0.97	0.97	0.97
Tomato_Tomato_mosaic_virus	0.97	1.00	0.98
Tomato_healthy	0.97	1.00	0.98
Weighted Avg	0.96	0.96	0.96

A consolidated comparison of the models was calculated as shown in **Table 9**. It can be seen that the combination of DNN features and LR performed substantially better than the other combinations in terms of accuracy, precision, recall, and F1-score. XGB+DNN produced good results as well, with RF+DNN and GB+DNN considerably more competitive. The poor performance of KNN DNN also supports the notion of selecting classifiers that would be suitable for the high-dimensional deep feature representations.

**Table 9:** Performance of hybrid ML+DNN models on plant disease dataset.

Model	Accuracy	Precision	Recall	F1 score
RF+DNN	0.9178	0.9216	0.9178	0.9171
XGB+DNN	0.9356	0.9392	0.9356	0.9348
GB+DNN	0.9022	0.9101	0.9022	0.9041
KNN+DNN	0.8089	0.8129	0.8089	0.8083
LR+DNN	0.9622	0.9624	0.9622	0.9621

In the order of theoretical measures, the analysis will be further supplemented with other studies that explain in more detail the workings of the hybrid models.

**Figure 3:**
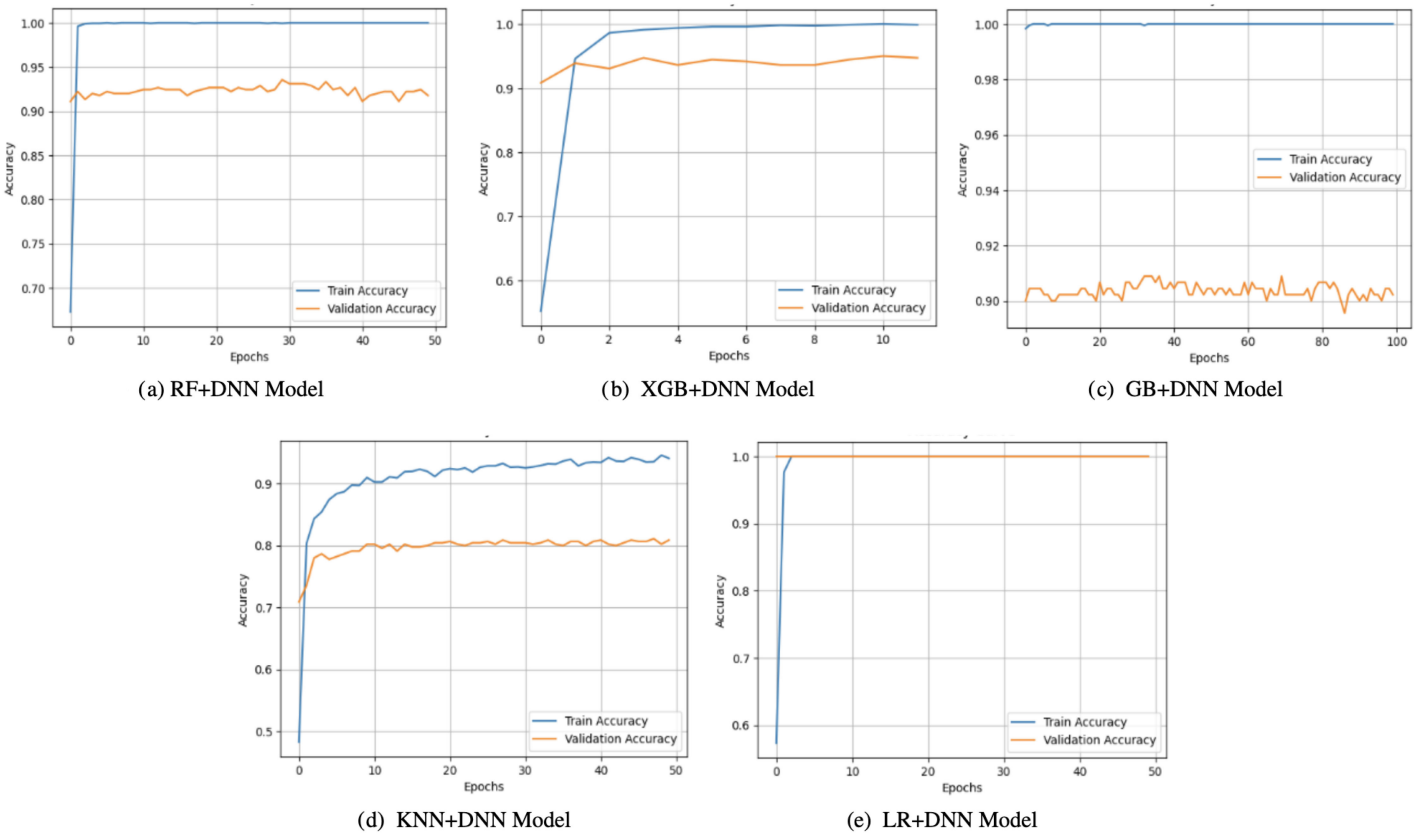
Accuracy curves of proposed models: **(a)** RF+DNN Model, **(b)** XGB+DNN Model, **(c)** GB+DNN Model, **(d)** KNN+DNN Model, and **(e)** LR+DNN Model.

Observations on the accuracy curves (**Figure 3**) indicated that there were changes between the generalization abilities of the hybrid models. On the one hand, both KNN+DNN and RF+DNN had increasing training accuracy over the epochs, but their validation accuracy exhibited errors and stagnation, implying that validation accuracy was prone to overfitting. Notably, KNN+DNN was highly dependent on the local data distribution, whereas RF+DNN even exhibited oscillatory behavior within validation. On the flip side, GB+DNN and XGB+DNN converged more smoothly, and validation accuracy closely mirrored training accuracy in the early epochs. However, because of training, small gaps opened up, which implied that minimal overfitting occurred later. LR+DNN achieved the most stable performance, with the accuracy of training and validation increasing simultaneously and remaining stable up to a certain point. Such linear agreement indicates the high level of generalization of the model that takes advantage of the simplicity of LR and the representational power of the DNN, allowing it to both represent linear dependencies and nonlinear ones.

**Figure 4:**
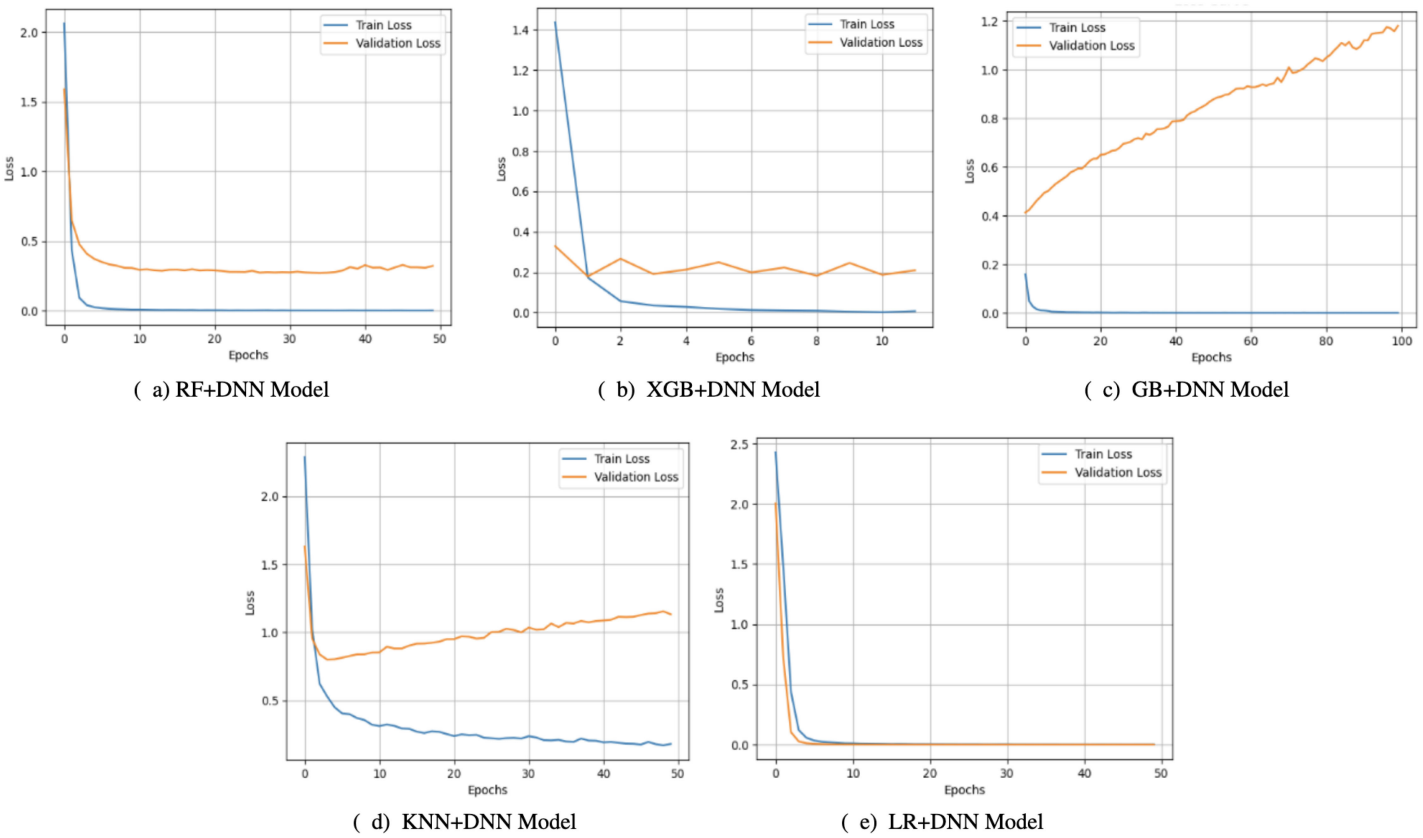
Loss curves of proposed models: **(a)** RF+DNN Model, **(b)** XGB+DNN Model, **(c)** GB+DNN Model, **(d)** KNN+DNN Model, and **(e)** LR+DNN Model.

The loss curves (**Figure 4**) also strengthened these observations. KNN+DNN and RF+DNN demonstrated smooth decreasing training loss. Yet, their validation curves became quite erratic and leveled off or even rose, which again attests to overfitting of the training data and lack of further optimization on the unseen samples. GB+DNN and XGB+DNN also showed a nicer decline in training and validation loss. However, slight differences emerged after epoch 500, suggesting that the models remained somewhat fragile regarding the complexity of boosting and the depth of optimization. In comparison, LR+DNN exhibited the most leveled-out behaviors, such that the training and validation loss reduced almost simultaneously across the epochs. The two curves did not show any significant differences, indicating that the model was not only effective in minimizing training error but also in transferring learning to the validation set, thereby signifying its robustness.

**Figure 5:**
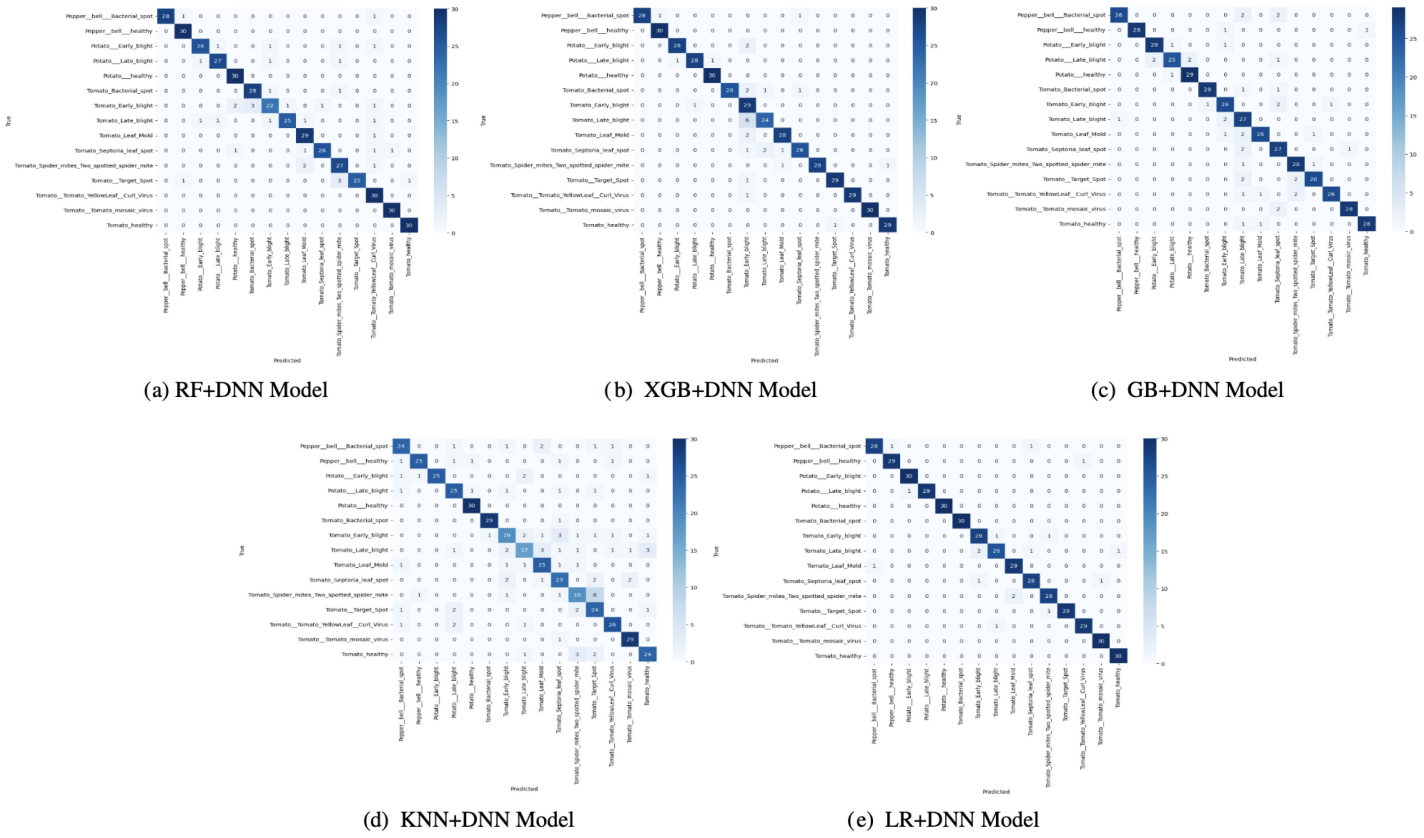
Confusion matrix of proposed models: **(a)** RF+DNN Model, **(b)** XGB+DNN Model, **(c)** GB+DNN Model, **(d)** KNN+DNN Model, and **(e)** LR+DNN Model.

The confusion matrices in **Figure 5** provide a detailed view of the success of the performance of each of the models in differentiating classes. The XGB+DNN and GB+DNN have been shown to exhibit good characteristics on the majority classes with strong diagonal dominance over frequent categories. Nonetheless, the two models did not deal well with minority classes, as their false negatives were high, proving a bias to well-represented samples. This indicates that, even though the boosting increased the predictive power, the skew in the data did not allow them to generalize to the entire set of classes. This was partially compromised in the RF+DNN model, as some enhancements in the visualization were related to the distribution of the classes. It had a better balance in its confusion matrix than boosting-based hybrids, but the misclassifications were non-trivially low, especially in the differentiation between closely related categories. The same can be said about KNN+DNN, which considers only the structure of local neighbors, thus being vulnerable to noise and overlapping classes, which contributed to the increased off-diagonal entries. The LR+DNN model was consistently proven to be superior in terms of confusion matrix structure, as evident from the apparent diagonal dominance in most classes and a significant reduction in off-diagonal errors. The fact that it can generate only a few false negatives, while also producing fewer false positives, illustrates its strength. This result indicates that LR+DNN is at once demonstrating greater overall accuracy but also predictive performance that is more fairly distributed across both more common and less common categories, a highly preferable property in real-world deployments in which imbalanced classes are prevalent.

**Figure 6:**
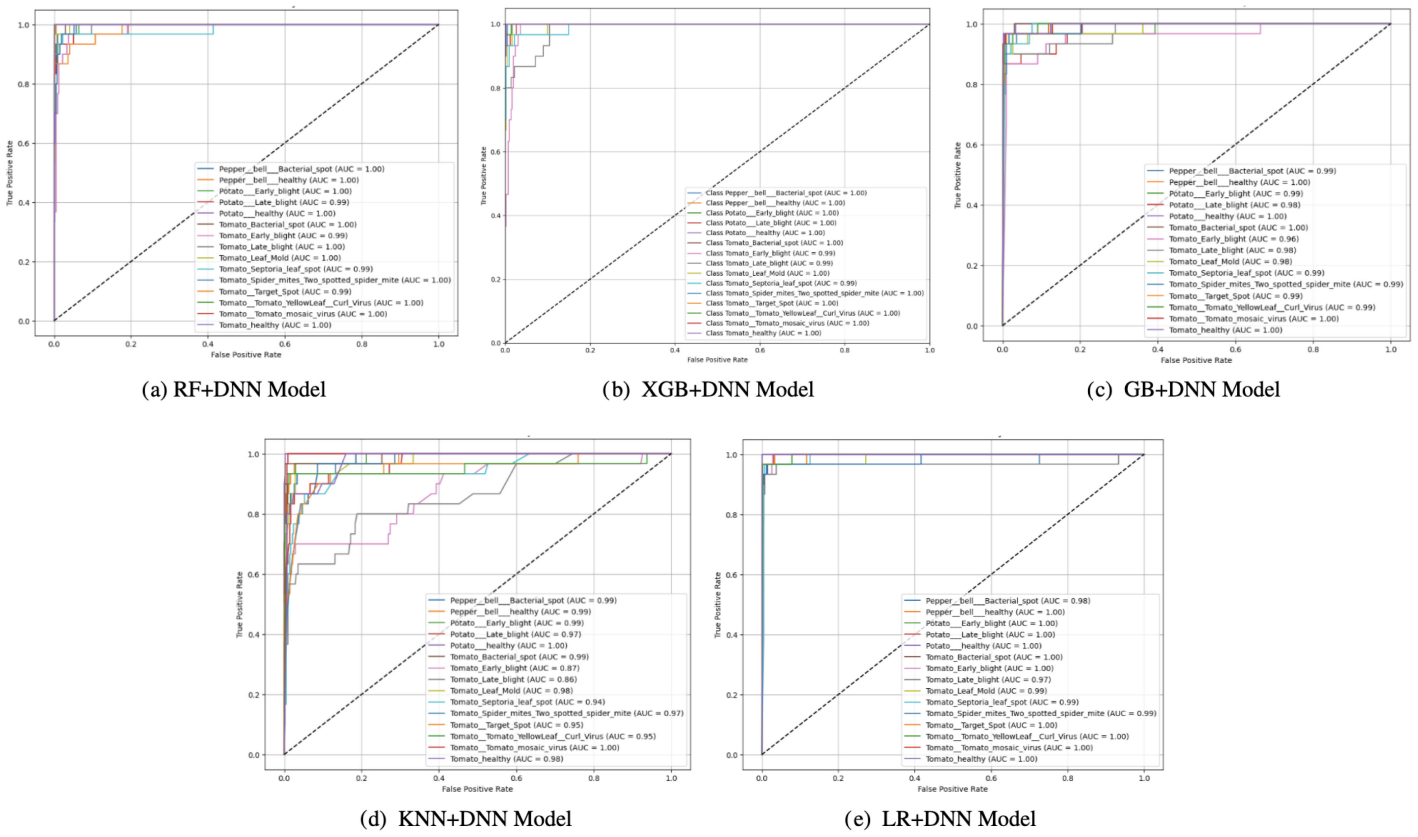
ROC curves of proposed models: **(a)** RF+DNN Model, **(b)** XGB+DNN Model, **(c)** GB+DNN Model, **(d)** KNN+DNN Model, and **(e)** LR+DNN Model.

ROC analysis (**Figure 6**) presents a global perspective of the discriminative ability of every model at different thresholds. Although all the models yielded decent results in terms of the ROC, differences in steepness of the curves and AUC underline that LR+DNN was superior to others. Models such as KNN+DNN and RF+DNN produced moderate ROC curves with less sharp increases, indicating their inability to find the optimal balance between sensitivity (true positive rate) and specificity (false positive rate). GB+DNN and XGB+DNN were viewed as superior due to their decent AUC rankings and sharper curves. However, their use was also constrained by their distributional varieties, which led to over-interpretation of minority classes, as reflected in the confusion matrices. However, the LR+DNN model showed that the steepest rise in the ROC curve was at the y-axis, and the AUC was the highest across all the models. This sharp slope indicates that the model rapidly achieves high sensitivity with minimal false positives, a crucial quality in model deployment for sensitive fields where false alarms are particularly problematic. The superior discriminating nature is substantiated by the high AUC, which attests that LR+DNN is very dependable regarding deciphering between positive and negative cases at all decision thresholds.

**Figure 7:**
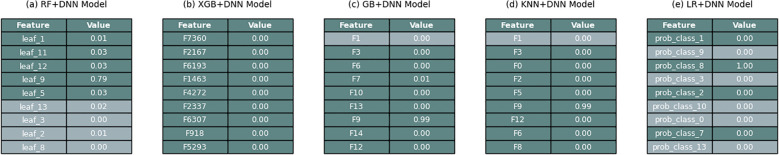
Local interpretable model-agnostic explanations of proposed models.

LIME (**Figure 7**) is more transparent because it highlights the most critical features in classification. In all models, LIME was found to focus on features obtained through PCA-reduction of ResNet embeddings as essential discriminators. Nevertheless, LR+DNN had the most intelligible and readable feature importance profiles. In contrast to such tree-based hybrids spreading weight over several less-informative features, LR+DNN focused on a few strong predictors and both the model output and the predictions were more aligned with what could be expected in the domain. This interpretability increases confidence in the results and offers practical information to future monitoring and diagnosis approaches of the disease.

The experimental findings clearly demonstrated that among the proposed hybrid models, the LR+DNN model outperformed the others across all evaluation metrics, establishing it as the most efficient model. In comparison, the XGB+DNN model secured the second-best performance, while the KNN+DNN model ranked lowest, indicating its limited suitability for the task at hand. A systematic evaluation of the models was conducted using a robust assessment framework that included the classification report, accuracy, precision, recall, F1-score, training history curves, confusion matrix, ROC analysis, and LIME for interpretability. This comprehensive analysis not only reinforces the superiority of the LR+DNN model but also provides valuable insights into the relative strengths and weaknesses of each model. Such clarity allows for a better understanding of their applicability in future tasks, thus guiding the selection of the most appropriate model for specific predictive challenges.

### 5.2 Discussion

In this paper, an optimized hybrid framework that uses pre-trained ResNet to extract features, as well as using the PCA and a combination of each of the ML classifiers (RF, XGB, GB, KNN, and LR) and corresponding dedicated DNN was introduced to detect plant diseases. By critically comparing the output of each of the models’ classification reports, learning curves, confusion matrices, ROC analysis, and LIME interpretability, we showed that in all the difficult plant disease classes, the LR+DNN model recorded the best stability and accuracy in general. In comparison to available literature especially the model proposed by (Haridasan et al., 2023) where authors used a combination of convolutional neural networks and support vector machines to detect five diseases in rice; it is worth mentioning that this model got an accuracy of 91.45% in validation, which is lower as compared to what our hybrid model using LR and DNN achieved with 96.2% accuracy as depicted in **Table 10**. In addition, our framework covered a wider range of disease classes in various crops (pepper, potato, tomato), and this exhibited better generalization to other species and disease types. Our model was effective in reducing feature redundancy to a significant extent by considering PCA. Additionally, the coupled ML and DNN framework enabled improved representation learning and classification stability.

**Table 10:** Accuracy comparison of existing work and proposed models.

Model	Accuracy (%)
CNN+SVM (Haridasan et al., 2023)	91.45
RF+DNN (Proposed)	91.77
XGB+DNN (Proposed)	93.56
GB+DNN (Proposed)	90.22
KNN+DNN (Proposed)	80.89
LR+DNN (Proposed)	96.22

Nevertheless, in addition to raw accuracy, the LR+DNN model demonstrated better learning dynamics. Coincidence of training and validation accuracy, the loss line of convergence is a representation of sound optimization and lower overfitting as opposed to the SVM-CNN method. Class-wise evaluations also support this strength; confusion matrices indicated that LR+DNN reduced false positive and false negative values in both the majority and minority classes, a degree of detail underlying the proper performance in the comparison-based research. Lastly, model explainability, as provided by LIME, resulted in more transparent and more interpretable feature attributions. This is particularly notable in the case of LR+DNN, which led to increased trust in its decisions, a quality that is often lacking in end-to-end DL models, such as those employed by (Haridasan et al., 2023).

Overall, the Hybrid LR+DNN has the advantage of being superior to the other methods in terms of its classification-related outcome, but also provides better stability, interpretability, and generalizability to various plant disease data papers. Such attributes strengthen its potential as a user-friendly tool in plant disease diagnosis in automated systems and especially in resource-poor agricultural environments.

### 5.3 Limitations

Although the suggested hybrid learning model has high predictive accuracy, it has several limitations that need to be taken into consideration to put the findings in perspective and inform future studies. The initial and most obvious limitation is the data set. Although popular in the study of plant pathology, the collection of images in PlantVillage has been collected under very controlled laboratory conditions, with uniform lighting background, and individual leaves photographed in isolation. These design decisions, although they would help reduce noise and standardize the input data, do not completely emulate the situation of the real world in agriculture. In the sphere, other foliage usually covers the leaf; it is prone to shadows or uneven lighting, and dust or soil particles, as well as different distances and positions are likely to be photographed using a consumer-grade smartphone camera. Therefore, the accuracy obtained in this experiment (96.22% with the help of LR+DNN on ResNet features) might not directly apply to the field, and the results might decline considerably when having more heterogeneous inputs.

The other limitation is related to the representation of diseases and the type of crops. Despite the size of the PlantVillage dataset, it is eventually limited to 14 crop species and 26 diseases, which creates a 38-label dataset. A smaller set was picked in this research, which included tomato, potato and pepper, thus the disease coverage is further reduced. Such a narrowness implies that trained models are only trained on a particular set of crops and diseases, and their use in other crops, new pathogens, or disease strains in the region is untested. In addition, there is an insufficient number of examples of early stages of the disease in the dataset, when visual signals are faint, unclear, or intersect with the indicators of abiotic stress. The lack of a large and diverse sample of such early stages of the disease may be a limiting factor to the use of the model in the timely intervention of the disease in the actual farming situation. This paper methodologically uses feature extraction with pre-trained ResNet models and then classification with hybrid machine learning algorithms. Although the given transfer learning method utilizes the representational potential of ResNet, it comes with its limitations. The original ResNet designs are not specifically designed to address plant pathology, but generic object recognition (like ImageNet classification). Consequently, some of the fine-grained visual features that are specific to the plant diseases might not be fully represented in the extracted features. Also, the fact that a static feature extractor is selected implies that the hybrid models are not trainable and may limit their capacity to learn representations corresponding to plant disease classification. Future studies can help to refine the convolutional backbone or use domain-specific designs that are more sensitive to texture, venation patterns and lesion morphology.

The other significant weakness is associated with generalizability and strength. The existing analysis was based solely on stratified sampling in the PlantVillage dataset. Whereas stratification provides a balanced distribution of training and testing, it is not a solution to the larger problem of domain shift between the laboratory and field conditions. Moreover, the data set has more than one image of a single leaf (taken in various orientations or positions). Even though efforts were made to rely on training and testing splits, it is still possible that there will be some feature redundancy across subsets of features, which will unnaturally inflate the estimates of performance. The accuracy of the suggested models in various real-life conditions cannot be assured without testing them on external, in-the-wild data. Lastly, the accuracy that is reported is high, but interpretability and aspects of practical deployment are not investigated in this study. The hybrid model gives good classification performance, but lacks in telling agronomists and farmers which visual symptoms are driving the predictions, which may be detrimental to its interpretability. Also, mobile deployment, particularly in terms of computational efficiency and scalability, which are relevant in real-world agricultural tasks, was not directly tested. Discussing these points, and incorporating bigger and more heterogeneous datasets measured in the real field would assist in moving away from the proof-of-concept research to the practical decision-support tools that can be used to support precision agriculture.

## 6 Conclusion and future scope

This study proposed hybrid ML and DNN networks that were found useful in the diagnosis of plant diseases using both handcrafted descriptors and deep ResNet-based features enhanced using PCA. Hybrid combinations of RF+DNN (91.78%), XGB+DNN (93.56%), GB+DNN (90.22%), KNN+DNN (80.89%) and LR+DNN (96.22%) had been thoroughly tested. The LR+DNN hybrid performed best and showed a high level of consistent robustness on the majority and minority classes. Its training and validation curves were well aligned, demonstrating the trustworthy convergence and a reduced chance of overfitting, whereas LIME analysis made the role of the features easy to understand. These findings validate the superior predictability efficiency of hybridization over independent models, and the LR + DNN set another level of performance and accuracy in automated detection of plant diseases. On the whole, the framework has represented an efficient, precise, and understandable agricultural diagnostic solution, which can prove greatly beneficial in early disease management and help address sustainable farming.

Based on the weaknesses of the existing study, it is possible to conduct several further research directions. First, it would be useful to extend the analysis to field-acquired datasets that have different backgrounds and lighting conditions and also overlap in leaves, to have more evidence on the generalizability of the model. The early-stage imagery of diseases and abiotic stress conditions should be incorporated to enhance the sensitivity of the model to subtle symptoms and the practical usefulness of the model in managing the disease on time. Methodologically speaking, future research may seek to focus on fine-tuning deep convolutional backbones or implementing domain-specific designs in plant pathology to achieve fine-grained lesion morphology and venation patterns further. Besides, it would be better to incorporate explainable AI methods, which would increase interpretability and credibility, allowing farmers and agronomists to be aware of the visual means by which the predictions are made. Lastly, exploring the implementation of lightweight hybrid models on mobile or edge devices would bridge the research and real-world gap in agricultural applications and therefore, automated plant disease detection would be more accessible and scalable in resource-constrained conditions.

## Data Availability

The original contributions presented in the study are included in the article/supplementary material. Further inquiries can be directed to the corresponding authors.
